# The Centrosomal Kinase Plk1 Localizes to the Transition Zone of Primary Cilia and Induces Phosphorylation of Nephrocystin-1

**DOI:** 10.1371/journal.pone.0038838

**Published:** 2012-06-11

**Authors:** Tamina Seeger-Nukpezah, Max C. Liebau, Katja Höpker, Tobias Lamkemeyer, Thomas Benzing, Erica A. Golemis, Bernhard Schermer

**Affiliations:** 1 Department of Developmental Therapeutics, Fox Chase Cancer Center, Philadelphia, Pennsylvania, United States of America; 2 Department II of Internal Medicine and Center for Molecular Medicine Cologne, University of Cologne, Cologne, Germany; 3 Department of Pediatrics, University of Cologne, Cologne, Germany; 4 Cologne Excellence Cluster on Cellular Stress Responses in Aging-Associated Diseases, University of Cologne, Cologne, Germany; Texas A&M University, United States of America

## Abstract

Polo-like kinase (Plk1) plays a central role in regulating the cell cycle. Plk1-mediated phosphorylation is essential for centrosome maturation, and for numerous mitotic events. Although Plk1 localizes to multiple subcellular sites, a major site of action is the centrosomes, which supports mitotic functions in control of bipolar spindle formation. In G0 or G1 untransformed cells, the centriolar core of the centrosome differentiates into the basal body of the primary cilium. Primary cilia are antenna-like sensory organelles dynamically regulated during the cell cycle. Whether Plk1 has a role in ciliary biology has never been studied. Nephrocystin-1 (NPHP1) is a ciliary protein; loss of NPHP1 in humans causes nephronophthisis (NPH), an autosomal-recessive cystic kidney disease. We here demonstrate that Plk1 colocalizes with nephrocystin-1 to the transition zone of primary cilia in epithelial cells. Plk1 co-immunoprecipitates with NPHP1, suggesting it is part of the nephrocystin protein complex. We identified a candidate Plk1 phosphorylation motif (D/E-X-S/T-φ-X-D/E) in nephrocystin-1, and demonstrated in vitro that Plk1 phosphorylates the nephrocystin N-terminus, which includes the specific PLK1 phosphorylation motif. Further, induced disassembly of primary cilia rapidly evoked Plk1 kinase activity, while small molecule inhibition of Plk1 activity or RNAi-mediated downregulation of Plk1 limited the first and second phase of ciliary disassembly. These data identify Plk1 as a novel transition zone signaling protein, suggest a function of Plk1 in cilia dynamics, and link Plk1 to the pathogenesis of NPH and potentially other cystic kidney diseases.

## Introduction

Cilia are microtubule-based sensory organelles projecting from the surface of almost all mammalian cells [1]. Covered by a specialized plasma membrane compartment, cilia play a major role in cell signaling during chemosensation and mechanosensation [2]. Ciliary assembly occurs in the G1 or G0 phases of the cell cycle, as the mother centriole converges to the plasma membrane and forms a basal body. This provides an anchor point for extension of the microtubule core of the cilium and positions the cilium at the apical membrane of the cell. Ciliary resorption prior to S-phase involves release of the basal body and subsequent centrosomal duplication in early S-phase [3]. Thus, the periodic reassembly and reabsorption of primary cilia are regulated by the cell cycle. Reciprocally, through signaling and sequestration of the mother centriole in the basal body, primary cilia influence cell cycle progression [4].

Primary cilia were discovered more than a century ago in the kidney, but were regarded as evolutionary remnants. However, numerous studies from the last decade have uncovered defects in cilia or cilia-affected proteins are linked to the pathogenesis of hereditary cystic kidney diseases, since almost all cystic disease-causing genes encode proteins localized in this specialized organelle [5]. Mutations of the human genes encoding the polycystins (PKD1 and PKD2) cause autosomal-dominant polycystic kidney disease (ADPKD), the most frequent polycystic kidney disease in adults, leading to end-stage renal disease in adulthood. Lov1, the *C. elegans* ortholog of polycystin-1, was first localized at neuronal cilia in 1999 [6]. Nephronophthisis (NPH) is the most frequent genetic cause of kidney failure in children and adolescents, associated with corticomedullary cysts and tubulointerstitial fibrosis. NPH is a genetically heterogeneous disease that may occur with an isolated renal phenotype or syndromic presentation. To date, 12 different genes (*NPHP1-12*) have been identified as mutated in patients with NPH [7].

20% of the mutations observed in NPH target the *NPHP1* gene, encoding nephrocystin-1 (NPHP1) [8]. NPHP1 localizes to the transition zone at the ciliary base [9], [10]. In *C. elegans,* the NPHP1 homolog *nph-1* is part of a protein complex that regulates basal body anchoring [11] and together with the NPHP4 homolog *nph-4* modifies the structure of the microtubule-based ciliary axoneme [12]. Recent data point to roles for NPHP1 and NPHP4 in signaling [13] and vesicular trafficking [14], [15]. Very recently, Sang et al. described the composition of three distinct NPH protein complexes functioning at different cellular sites [7]. The overall function of NPH proteins and their role at the transition zone of primary cilia remains incompletely understood.

Although a number of studies link ciliary protrusion, resorption, and signaling to cell cycle control [4], it was only recently established that canonical regulators of centrosomes and mitosis can regulate non-mitotic functions of cilia. For example, the Aurora-A kinase (encoded by *AURKA*) regulates mitotic entry and exit, but is also crucial for ciliary resorption [16]. Since cells typically have to resorb their cilium to re-enter cell cycle, a defect in ciliary disassembly could be a second way in which defects in Aurora-A function affect proliferation and tissue repair. More recently, a second non-mitotic cilia-associated function of Aurora A has been observed, as Aurora-A was shown to phosphorylate and inactivate polycystin-2 (PKD2), a Ca(2+)-permeable nonselective cation channel localized in the ER and in cilia, and Aurora-A expression was found to be upregulated and activated in the renal cysts of human PKD patients [17], [18]. These unexpected direct interactions, which position Aurora-A, to regulate renal cell ciliary integrity and calcium signaling in a cystic disease, led us to investigate potential roles for other mitotic regulators.

During mitosis, Aurora A physically and functionally interacts with polo-like kinase 1 (encoded by *PLK1*). At the centrosome, the mitotic spindle, and the midbody, Plk1 phosphorylates substrates including ninein-like protein (NLP; also known as NINL), kizuna, the kinetochore-associated checkpoint kinase BUBR1 (also known as BUB1β) and the central spindlin complex protein, CYK4, that support mitotic progression (reviewed in [19], [20], [21], [22]). One interesting recent study demonstrated a function for Plk1 in microtubular dynamics [23]. In this study, we for the first time identify Plk1 as a ciliary protein localized at the transition zone of primary cilia in ciliated, non-mitotic cells. Moreover, we demonstrate an association of Plk1 with the nephrocystin protein complex and describe Nephrocystin-1 as a Plk1 substrate, and show that Plk1 activation occurs in ciliary disassembly. This is the first study linking Plk1 with primary cilia and with the NPH protein complex suggesting a potential function of Plk1 in the pathogenesis of the cystic kidney disease NPH.

## Materials and Methods

### Cell Culture, Transfection, Plasmids, siRNA and Inhibitors

HEK293T cells (received from American Type Culture Collection) [9] HEK293 and hTERT-RPE1 cells (received from Clontech Laboratories, Inc.) [15] were maintained in DMEM with 10% FBS. The immortalized human kidney proximal tubular cell line HK-2 (catalog no. CRL-219; American Type Culture Collection) were maintained in keratinocyte media supplemented with 0.05 mg/ml bovine pituitary extract and 5ng/ml recombinant EGF (Invitrogen). Transient transfection of HEK293 cells with expression constructs for PLK1, NPHP1 a panel of NPHP1 derivatives, and the negative interaction control protein EPS-1-225, was performed using the calcium phosphate method as previously described [24] or with Lipofectamine and Plus reagent (Invitrogen) according to the manufacturer’s instructions. *NPHP1* was cloned from a human kidney cDNA library into a modified pcDNA6 vector using standard PCR cloning techniques and has been described previously [9], [25]. *NPHP1* truncations were cloned by standard techniques and have been described [15]. The NPHP1 T87A mutation was introduced by site-directed mutagenesis (Quikchange, Stratagene, forward primer, AGAAAAGAAGAGGAGCATGCTCTTTTGGACAAACTTACCCAA and reverse primer TTGGGTAAGTTTGTCCAAAAGAGCAT GCTCCTCTTCTTTTCT were used). PLK1 pcDNA6 and FLAG.EPS-1-225 was cloned from a human podocyte cDNA library into a modified pcDNA6 vector. The siRNA ON-Target plus SMARTpool to Plk1 was used to deplete Plk1 (Thermo Scientific Dharmacon). The PLK1 inhibitor BI 2536 (Selleck Chemicals, Houston, TX) was used at a final concentration of 100 nM, diluted from a 10mM stock solution prepared in DMSO [26].

### Immunofluorescence

Cells were plated on coverslips in serum-free medium (OPTIMEM, Invitrogen) and grown for 48 hours to induce cilia formation, followed by treatments described in results. Cells were fixed with 4% paraformaldehyde for 5 min and cold methanol for additional 2 min. Cells were permeabilized with 0.1% Triton X-100 in PBS for 10 min, blocked in PBS with 3% BSA, and incubated with antibodies using standard protocols. Confocal microscopy was performed using a confocal microscope (C1 Spectral; Nikon) equipped with an NA 1.40 oil immersion 60x Plan Apochromat objective (Nikon). Images were acquired at room temperature using EZ-C1 3.8 (Nikon) software. Adjustments to brightness and contrast were minimal and were always applied to the whole image. For immunofluorescence, primary antibodies included mouse and rabbit anti-acetylated α-tubulin (clone 6-11B-1 [Sigma-Aldrich] and clone K(Ac)40 [Enzo Life Sciences]), rabbit anti-γ-tubulin (Sigma), mouse anti-γ-tubulin conjugated with Dy647 (Abcam), mouse anti-NPHP1 [15], rabbit anti-PLK1 (Novus Biologicals), and mouse anti-PLK1 phosphoT210 (Abcam). Secondary antibodies labeled with Alexa Fluor 488 (goat) or Alexa Fluor 568 (goat), and DAPI to stain DNA were obtained from Invitrogen.

### Protein Expression, Western Blotting, and Immunoprecipitation

Recombinant His-tagged NPHP1 1-205 and His-tagged NPHP1 237–670 were bacterially expressed in BL-21 CodonPlus RIPL (Stratagene) and affinity-purified by standard methods. For western blotting and immunoprecipitation, mammalian cells were disrupted in lysis buffer (CelLytic M; Sigma-Aldrich) supplemented with a protease and phosphate inhibitor cocktail (Thermo Scientific). Whole-cell lysates were used either directly for SDS-PAGE or for immunoprecipitation. Immunoprecipitation samples were incubated for 2 h with anti-Flag M2 affinity gel (Sigma-Aldrich), washed and resolved by SDS-PAGE (Nu-PAGE, Invitrogen). To analyze the expression level of PLK1 and phospho-PLK1 cells were collected by centrifugation and directly resuspended in sample buffer (Invitrogen) before being resolved by SDS-PAGE. For Western Blot analysis, primary antibodies included mouse anti-Myc (9E10, Santa Cruz), mouse anti-Flag (M2, Sigma-Aldrich), mouse anti-V5 (Millipore), rabbit anti-PLK1 (Cell Signaling), rabbit anti-PLK1 phospho-T210 (Cell Signaling), and mouse anti-β-actin conjugated to HRP (abcam). Secondary anti-mouse and anti-rabbit HRP-conjugated antibodies (GE Healthcare) were used at a dilution of 1∶10,000. Western Blotting was performed using standard procedures, and developed by chemoluminescence using Super Signal West Pico and Femto substrate (Thermo Fisher Scientific). Quantification of signals on Western blots was done using the NIH ImageJ Imaging and Processing Analysis Software with signaling intensity normalized to β-actin, and calculated for 3 independent experiments. Statistical analysis was performed by t-test.

### Kinase Assay

To assess phosphorylation of NPHP1 truncations by PLK1, an *in vitro* kinase assay was performed using bacterially expressed His-tagged NPHP1 1–205, His-tagged NPHP1 237–670 and recombinant active Plk1 (Abcam) in a buffer containing 20 mM Tris–HCl, 50 mM KCl, 10 mM MgCl2, pH 7.5, 100 µM ATP, with or without γ-[32P]ATP [9]. Parallel aliquots without γ-[32P]ATP were processed for SDS-PAGE/Coomassie blue staining.

### Ciliary Disassembly Assay

hTert-RPE1 cells were plated on glass cover slips in serum-free medium (OPTIMEM, Invitrogen) and starved for 48 hours to induce cilia assembly. Two to three hours prior to the initiation of ciliary disassembly cells were incubated with PLK1 inhibitor BI 2536 at 100 nM, or with vehicle DMSO. Ciliary disassembly was induced with DMEM and 10% FBS [16]. At the time of induction of disassembly, and after 2, 6, and 24 hours, cells were fixed and stained for acetylated-α-tubulin and γ-tubulin to visualize cilia and axoneme. An average of 250 cells per condition was counted in three independent blinded experiments. Assessment of cilia length was done using the NIH ImageJ Imaging and Processing Analysis Software measuring an average of 50 cilia per condition of 3 independent experiments. T-testing was used for statistical analysis.

### Tryptic In-gel Digest

SDS-PAGE bands of interest were digested as described elsewhere [27]. In brief, bands were cut out and minced. After destaining with 50% 10 mM NH4HCO3/50% CAN at 55°C and dehydration in 100% CAN, gel pieces were equilibrated with 10 mM DTT in 10 mM NH4HCO3 for 45 minutes at 56°C. After subsequent dehydration with 100% CAN, gel pieces were incubated with 55 mM iodoacetamide in 10 mM NH4HCO3 for 10 minutes at room temperature in the dark for carbidomethylation of sulfhydryl groups. Gel pieces were again dehydrated (100% CAN) and then equilibrated with 10 mM NH4HCO3 containing porcine trypsin (12.5 ng/µl; Promega, Mannheim) on ice for 2 hours. Excess trypsin solution was removed and hydrolysis was performed for 4 hours at 37°C in 10 mM NH4HCO3. Digests were acidified with 5% TFA and the gel pieces were extracted twice with 0.1% TFA and then with 60% CAN/40%H2O/0.1% TFA, followed by a two-step treatment using 100% CAN. Extractions were combined, concentrated by vacuum centrifugation and desalted according to Rappsilber et al. [28].

### Nano-LC ESI-MS/MS Mass Spectrometry

Sample analysis was performed on a LTQ Orbitrap Discovery mass spectrometer (Thermo Scientific) that was coupled to a Proxeon EASY-nLC II nano-LC system (Proxeon/Thermo Scientific). Intact peptides were detected in the Orbitrap at 30,000 resolution in the mass-to-charge (m/z) range 200–2000 (MS). Internal calibration was performed using the ion signal of (Si(CH3)2O)6H at m/z 445.120025 as a lock mass. For LC-MS/MS analysis, up to ten CID spectra (MS2) were acquired following each full MS scan. Aliquots of the sample were separated on a 10 cm, 75 µm reverse phase column (Proxeon/Thermo Scientific). Gradient elution was performed from 10 to 40% acetonitrile within 120 minutes at a flow rate of 250 nl/min.

### Peptide and Protein Identification

Sequest was used for protein identification (including the phosphoRS node, which provides a confidence measure for the localization of phosphorylation in peptide sequences) (Proteome Discoverer 1.3, Thermo Scientific). For intact peptide masses, a 10 ppm mass tolerance was allowed and 0.8 Da for CID fragment ions detected in the linear ion trap. Data were searched using the canonical complete proteome sequence database of Homo sapiens (www.uniprot.org). The truncated sequence of human NPHP1 including N-terminal tags, as well as a sequence with an exchange of a threonine to an alanine residue at position 87 of the native protein sequence was copied into the database. Oxidation of methionine residues and phosphorylation of serine, threonine and tyrosine residues were used as variable and carbamidomethylation of cysteine residues as fixed modifications. For identification of peptides, search results were filtered for rank 1 peptides of medium or high confidence with a mass accuracy of ≤5 ppm, and containing at least six amino acid residues. The minimal score was set to 2.0 for charge state 2+, 2.25 (3+) and 2.5 (4+), respectively. In case of phosphopeptides, unfiltered search result data were analyzed manually.

## Results

### Plk1 Localizes at the Transition Zone of Primary Cilia

**Figure 1:**
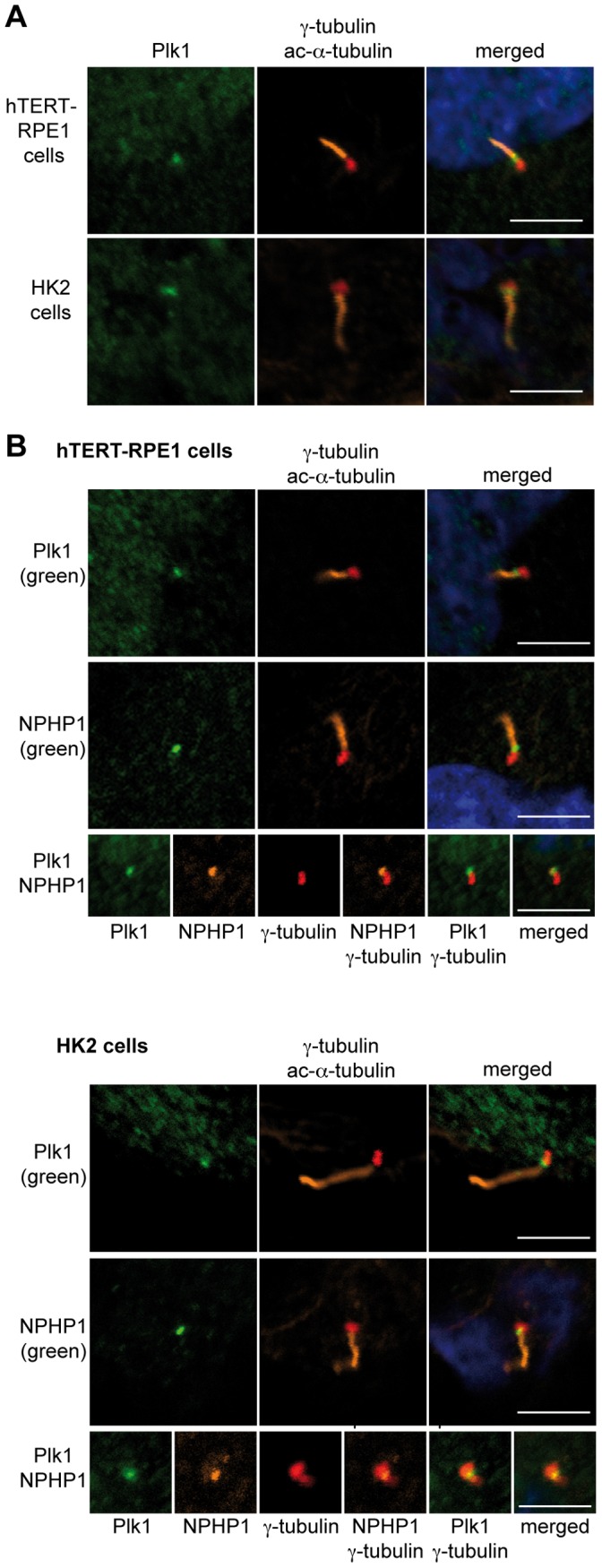
Plk1 colocalizes with NPHP1 at the base of the cilium. **A** Ciliated hTERT-RPE1 (human retinal pigmented epithelial cells and HK2 human kidney cells were stained with antibody to Plk1 (green), acetylated α-tubulin (orange), and γ-tubulin (red), and treated with DAPI to visualize DNA (blue). The scale bar represents 5 µm. **B** Ciliated hTERT-RPE1 cells and HK2 cells were stained with antibody to acetylated α-tubulin (orange), γ-tubulin (red), to NPHP1 or Plk1 as indicated (green), and with DAPI to visualize DNA (blue). The third row shows merged signals from staining with antibody to Plk1 (orange), NPHP1 (green) acetylated α-tubulin (orange), γ-tubulin (red) and DAPI was used to visualize DNA (blue). The scale bar represents 5 µm.

We first examined the localization of Plk1 in two different human cell lines, HK-2 and hTERT-RPE1, which develop primary cilia following serum starvation and maintenance under high confluence conditions. In both cell lines, Plk1 localized to the base of primary cilia (Fig. 1A). Interestingly, although Plk1 usually co-localizes with γ-tubulin at centrosomes in cycling cells [29] (Fig. S1), the Plk1 signal did not overlap with the γ-tubulin signal marking the basal bodies, but was consistently slightly displaced into the region between the basal body and the ciliary shaft, which reflects the transition zone [30]. Plk1 strikingly colocalized with the transition zone protein nephrocystin-1 (NPHP1) in both cell lines (Fig. 1B).

### Plk1 is a Novel Component of a Nephrocystin Protein Complex

**Figure 2:**
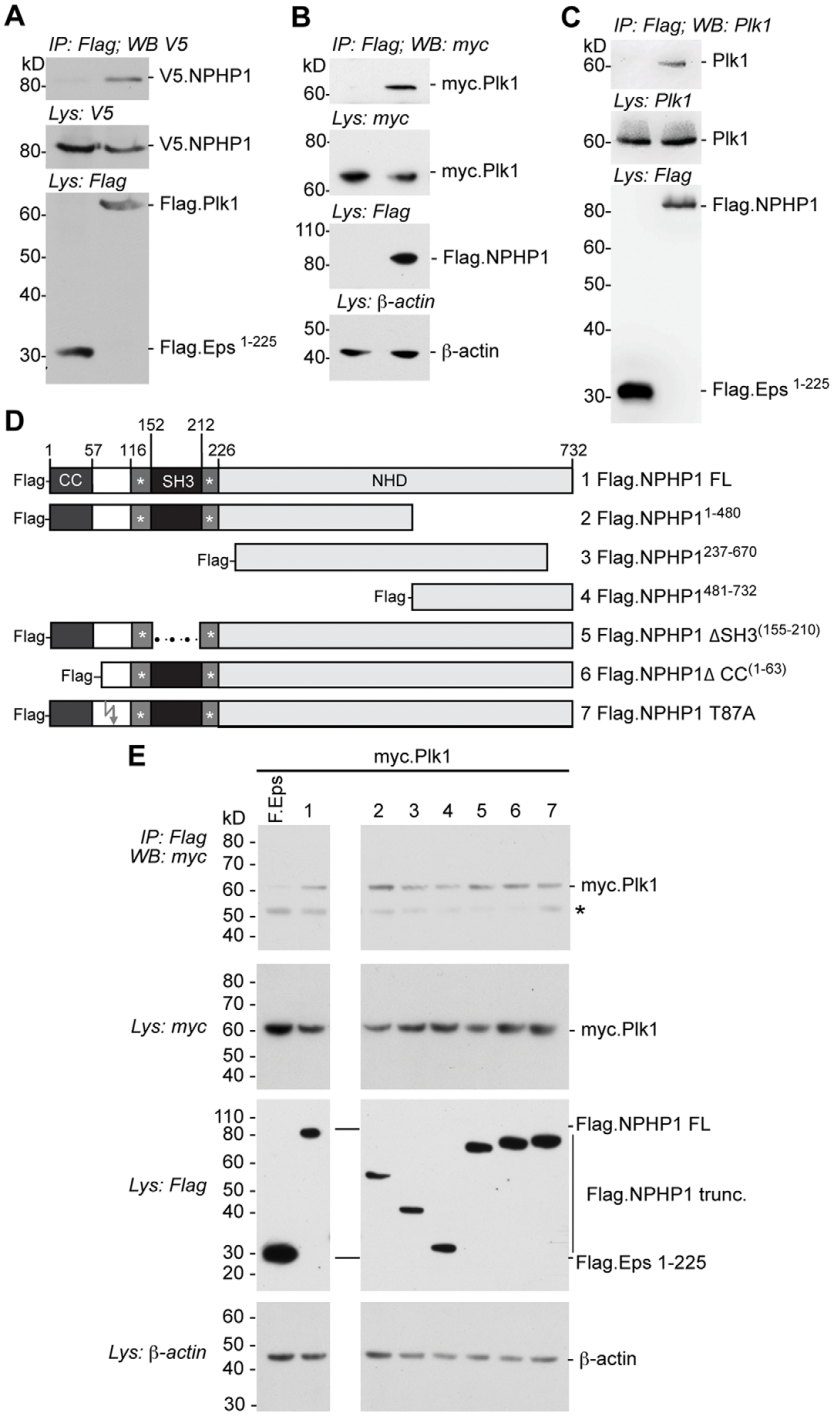
Plk1 associates with NPHP1. **A** Western blot of immunoprecipitates (IP) or lysates (Lys) from HEK293T cells co-transfected with plasmids expressing V5-tagged NPHP1 and Flag-tagged Plk1 or negative control protein (Eps1–225 [13]). β-actin was assessed as a loading control. **B** Western blot of immunoprecipitates (IP) or cell lysates (Lys) from HEK293T cells co-transfected with plasmids expressing Myc-tagged Plk1 and Flag-tagged NPHP1 or empty Flag vector. **C** Western blot of immunoprecipitates (IP) or cell lysates (Lys) from HEK293T cells transfected with plasmid expressing Flag-tagged NPHP1 or the negative control protein (Eps1–225 [13]). Endogenous Plk1 was detected using a specific antibody against Plk1. **D** A panel of Flag-tagged NPHP1 derivatives, including truncations, internal deletions and a T87A mutant, was analyzed by co-immunoprecipitation with Myc-tagged Plk1. **E** Western analysis of immunoprecipitates (IP) or cell lysates (Lys) from HEK293T cells co-transfected with plasmids expressing Myc-tagged Plk1 and Flag-tagged NPHP1 constructs as indicated, or the Flag-tagged control protein (Eps1–225). * indicates immunoglobulin heavy chain.

Since Plk1 colocalizes with NPHP1, we used a co-immunoprecipitation approach to assess whether these proteins interacted. V5.NPHP1 was coexpressed with either Flag.Plk1 or Flag.EPS-1-225 (as a negative control protein) in HEK293T cells. Precipitation of FLAG-tagged proteins with M2 beads showed that V5.NPHP1 specifically co-precipitated with Flag-Plk1 (Fig.2A). In the reverse experiment, myc.PLK1 was co-expressed with either Flag.NPHP1 or with the empty Flag-containing vector. Again, Plk1 co-precipitated with Flag.NPHP1 (Fig.2B). Similarly, endogenous PLK1 co-precipitated with Flag.NPHP1 but not with the control protein Flag.EPS-1-225 (Fig.2C).

NPHP1 has an N-terminal coiled-coil domain (CC) and a Src-homology 3 domain (SH3), flanked by acidic clusters. To map the area of Plk1-NPHP1 interaction we co-transfected myc-Plk1 with truncated derivatives of NPHP1 lacking the N- or C-terminus or other evolutionarily conserved domains (Fig. 2C). All truncations co-precipitated myc.Plk1 in these experiments (Fig. 2D), although less myc.Plk1 was pulled down by constructs Flag.NPHP1-237-670 and Flag.NPHP-481-732. The fact that non-overlapping truncations interacted and that the interactions could not be mapped to a single domain suggests that the interaction of Plk1 and NPHP1 might be mediated by a common interaction partner rather then being a direct protein-protein interaction, or alternatively, that Plk1 and NPHP1 interactions involve multiple sites on each protein.

### Recombinant Plk1 Phosphorylates NPHP1

**Figure 3:**
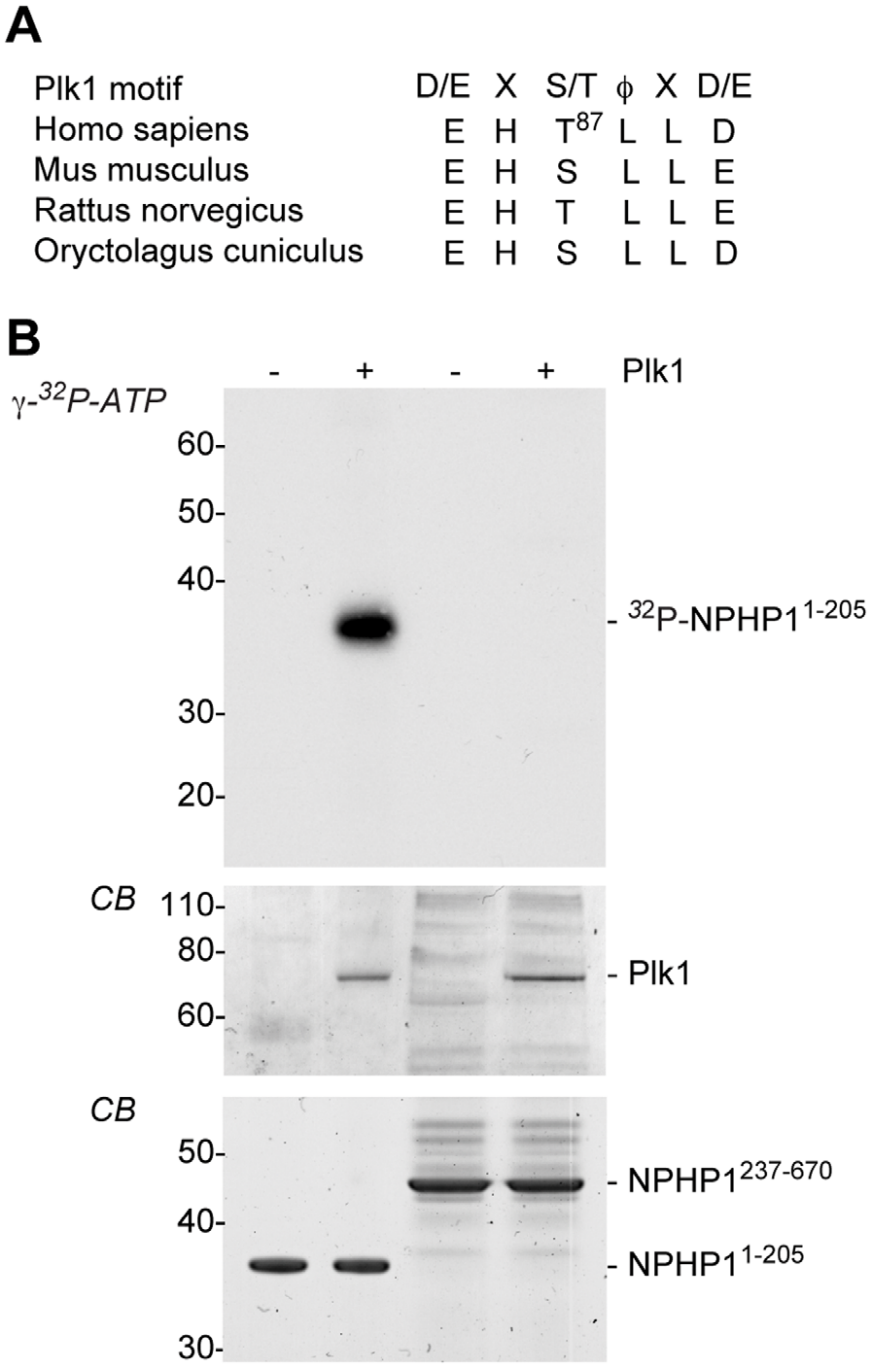
Plk1 directly phosphorylates NPHP1 in vitro. **A** Alignment of NPHP1 protein sequences from multiple species indicates a conserved candidate Plk1 motif at position T87. **B** An *in vitro* kinase assay performed with active Plk1 and recombinant His-fused NPHP1 protein indicates phosphorylation within the NPHP1 N-terminal 205 amino acids. CB, Coomassie Blue.

We next assessed whether Plk1 directly phosphorylates NPHP1. Strikingly, NPHP1 contains a conserved candidate Plk1 phosphorylation motif [31] within its N-terminus (res 85–90, EHTLLD, between the CC and SH3 domains) (Fig. 3A). To test whether NPHP1 might be a Plk1 substrate, two bacterially expressed His-tagged NPHP1 truncations were affinity purified and used for an *in vitro* kinase assay with recombinant active Plk1. The N-terminal truncation containing the Plk1 motif His.NPHP1-1-205 was highly phosphorylated by Plk1, while His.NPHP1-237-670 was not (Fig. 3B). Thus, the function of Plk1 at the ciliary transition zone could be related to the phosphorylation of NPHP1. To test whether the conserved phosphorylation motif at residue T87 can be directly phosphorylated by Plk1, we performed mass spectrometry following *in vitro* kinase assay with active Plk1 and assessed His.NPHP1-1-205, versus His.NPHP1-1-205(T87A), which eliminates the conserved Plk1 phosphorylation motif. His.NPHP1-1-205 was directly phosphorylated by Plk1 on the phosphorylation motif, while His.NPHP1-1-205(T87A), lacking the motif at residue 87, failed to be phosphorylated (Fig. S4).

### Plk1 and the Disassembly of Primary Cilia

**Figure 4:**
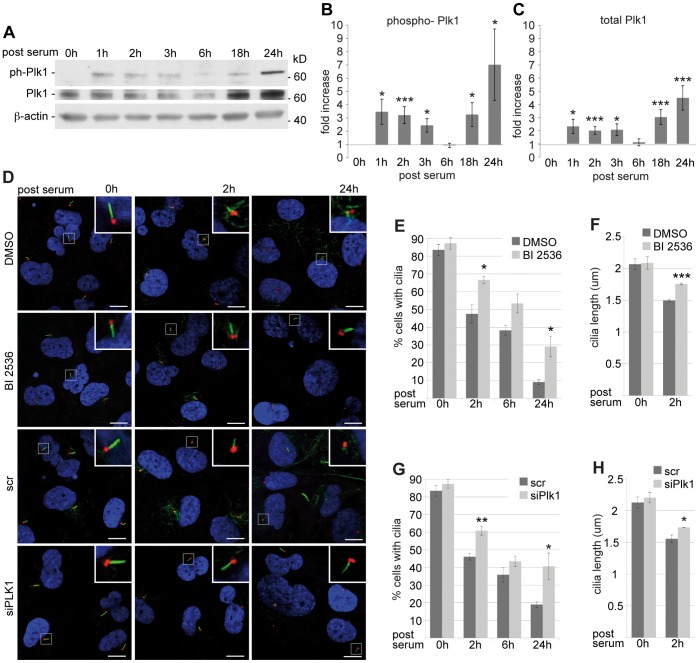
Plk1 is activated during ciliary disassembly but has limited influence on disassembly dynamics. **A** Immuno blot analysis of whole cell lysates prepared from hTERT-RPE1 grown under starved, ciliated conditions (0h) or at the times indicated following serum treatment to induce ciliary resorption. Ph-Plk1^T210^ represents activated Plk1; **B, C** Quantification of the expression of (**B**) Ph-Plk1^T210^ or (**C)** total Plk1 normalized to β-actin and relativized to the expression level without serum induction. Results from three independent experiments were calculated. * P<0.05, ***P≤0.005. Error bars represent SE. **D** Upper panel: Immunofluorescence of starved ciliated hTERT-RPE1 cells treated with the Plk1-inhibitor BI 2536 or with DMSO for 3 hours, then maintained in serum-free medium, or induced for ciliary disassembly with 10% serum-containing medium. Lower panel: Immunofluorescence of serum-starved, ciliated hTERT-RPE1 cells treated with siRNA to Plk1 or with scrambled (scr) control siRNA after 0, 2 and 24 hours of serum addition. Image shows acetylated α-tubulin (green), γ-tubulin (red) and DNA (blue). The scale bar represents 10 µm. **E** - **H** Quantification of three independent repetitions of experiment presented in **D**. **E** and **G** Quantification of the percentage of ciliated cells, with an average of 250 cells counted for each condition. **F** and **H** Quantification of cilia length, with an average of 50 cilia measured for each condition. * P<0.05, **P<0.01. ***P<0.001. Error bars represent SE.

In ciliated hTERT-RPE1 cells, addition of serum promotes two different waves of ciliary disassembly, at ∼2 hrs and ∼24 hrs after serum stimulation [16]. The early phase of cilia disassembly does not overlap with the re-entry into mitosis, but occurs in G0/G1 populations [16]. We assessed whether Plk1 is activated during the serum-induced ciliary disassembly in hTert-RPE1 cells. We found a highly significant increase of Plk1 phosphorylation (reflected by appearance of phosphorylated Plk1-pT210) after 1 hr of serum-induced ciliary disassembly, with this species persisting for 3 hours (Fig.4 A,B). After 6 hours, the levels of active Plk1 were consistently low, similar to the levels in serum starved cells, while Plk1 was reactivated at 18–24 hours after serum induction, corresponding to the typical time of entry of the cells into mitosis. Total Plk1 levels increased and decreased in parallel to the active pool of Plk1, but with variance over a more limited dynamic range (Fig. 4B, C).

To confirm our hypothesis that Plk1 activity directly influences ciliary disassembly, ciliated, serum starved hTERT-RPE1 cells were treated for 3 hours with the specific Plk1 inhibitor BI 2536 (100 nM ) versus vehicle DMSO before ciliary disassembly was induced by adding serum. The inhibition of PLK1 modestly but consistently inhibited the disassembly process, as shown by the reduced ciliary resorption of BI 2536-treated cells compared to control cells (87.3% versus 83.5% ciliated cells at the starting point, but 66.6% versus 47.5% ciliated cells remaining at 2 hours (p<0.05), and 53.4% versus 38.2% at 6 hours, 29.1% versus 9.1% ciliated cells at 24 hours after induction of ciliary disassembly (p<0.05) (Fig.4D, E)). BI 2536 treatment also increased the length of residual cilia (2.09 µm versus 2.07 µm cilia length at 0 hours, but 1.76 µm versus 1.49 µm at 2 hours (p<0.001) (Fig.4D, F)).

To further confirm a specific effect of Plk1 inhibition, cells plated in serum-free medium were transfected with siRNA to Plk1 or control siRNA (scr) for 48 hours, before ciliary resorption was analyzed. In similar results, the percentage of ciliated cells was comparable in serum-free medium (87.4% versus 83.5% ciliated cells), but cilia resorption was inhibited by knockdown of Plk1 (60.9% versus 46.1% ciliated cells remaining at 2 hours (p<0.001), 43.5% versus 35.9% at 6 hours, and 40.6% versus 18.9% ciliated cells at 24 hours after serum induction (p<0.05) (Fig.4D, G; confirmation of the knockdown was shown by immuno blot Fig. S3)). Length of residual cilia was again increased in cells with reduced Plk1 levels (2.20 µm versus 2.13 µm cilia length in serum-starved cells and 1.74 µm versus 1.56 µm at 2 hours after addition of serum (p<0.05) (Fig.4D, H)).

Using immunofluorescence, we assessed the localization of total and active Plk1 during the two waves of ciliary resorption (Fig. S1). Total Plk1 localized comparably to the transition zone both in serum-starved cells and at 2 hours after serum induction, in ciliated and non-ciliated cells. At 24 hours Plk1 could be also found at the transition zone of some of the few remaining cilia, although the dominant locus of PLK1 expression at this time point was in the mitotic centrosomes, spindle, and midbody, as expected. In contrast, active Plk1 (ph-Plk1 T^210^) was much less abundantly found in serum-starved cells at the transition zone while it was commonly seen at the transition zone of both ciliated (42%) and deciliated (58%) cells two hours after serum induction (Fig. S1). Suggestively, cilia staining positive for phospho-Plk1 at the transition zone were typically shorter than cilia without similarly localized active Plk1 (1.2 µm versus 1.9 µm; p<0.003), supporting the idea that activation accompanies resorption. At 24 hours after serum stimulation, the localization profile of active Plk1 parallels that of total Plk1 at the mitotic apparatus (Fig. S1).

Finally, NPHP1 localizes comparably to the transition zone, proximal to the basal body or centrosome, both in serum-starved cells and at 2 hours and 24 hours after serum treatment, in both ciliated and non-ciliated cells (Fig. S2). To investigate the effect of Plk1 activity on NPHP1 localization in ciliated cells and during the disassembly process, we treated cells either with the Plk1 inhibitor BI 2536 versus DMSO, or transfected siRNA against Plk1 versus scrambled siRNA (scr), paralleling the disassembly experiments above. The localization of total NPHP1 was not affected by either inhibition or depletion of Plk1 (Fig. S2).

## Discussion

Our study connects activation and function of the mitotic kinase Plk1, a major regulatory enzyme of mitotic processes, with primary cilia disassembly. Plk1 activation occurred shortly after induction of ciliary disassembly by serum stimulation, while in addition, we observed a subtle but significant reduction in the early phase of ciliary disassembly in presence of a specific inhibitor of Plk1. An earlier study has shown that neither knockdown nor inhibition of Plk1 significantly influenced the level of primary cilia formation [32], which we confirmed in our experiments (Fig.4D-H), indicating Plk1 functions specifically in disassembly.

The regulation of ciliary disassembly has recently been linked to another mitotic kinase, Aurora-A, which is activated by HEF1 at the ciliary basal body and promotes ciliary disassembly by activating the histone deacetylase HDAC-6 [16]. Interestingly, there is a close connection between Aurora-A and Plk1 in mitosis. During late G2 phase, Aurora-A activates Plk1 [33], while Plk1 targets Aurora-A to the centrosome [34], [35] and promotes Aurora-A degradation in late anaphase [36]. Potentially, Plk1 and Aurora-A may also interact at the ciliary basal body, and the subtle reduction in ciliary disassembly through inhibition of Plk1 might be either a direct Plk1 effect or mediated by the Aurora-A/HDAC-6 pathway.

Centrosomes are among the main subcellular localization sites of Plk1 [37], [38] and one of the main cellular functions of Plk1 is the regulation of centrosome maturation in concert with Aurora-A [39], [40]. Interestingly, the ciliary fraction of Plk1 was not primarily found at the basal body, which is directly built from the mother centriole. Instead, Plk1 co-localized with nephrocystin-1 at the transition zone, the region connecting the basal body, the ciliary axoneme and the plasma membrane. The transition zone has been implicated to function as ciliary gatekeeper controlling trafficking of ciliary proteins [41], [42] and many transition zone proteins including the nephrocystins have been suggested to be involved in this function [11], [43], [44]. In our study, Plk1 induced the phosphorylation of the transition zone protein nephrocystin-1. By phosphorylation of transition zone proteins Plk1 might modulate trafficking required for ciliary maintenance or disassembly. Interestingly, recent studies revealed that protein phosphorylation is a key event in the regulation of ciliary disassembly in Chlamydomonas and other model organisms [45], [46]. Therefore, inhibition of Plk1 induced phosphorylation of ciliary proteins could be another direct mechanism for the effect on ciliary disassembly we observed.

A number of recent studies identified targets of Plk1 that indicate functions in cellular processes beyond mitosis including microtubule dynamics, DNA replication, p53 regulation and the recovery from the DNA damage checkpoint (reviewed in [47]). A large number of putative substrates were identified through screens analyzing the interactome of the polo-box domain of Plk1 [48], and the Plk1-dependent phosphoproteome of the early mitotic spindle [49] and of mitotic human cells [50]. Taken together, these studies implicate that the mitotic kinase Plk1 is associated with many functions beyond the regulation of the core process of mitosis; it is intriguing to speculate whether one of these functions might be associated with the ciliary pool of Plk1.

Further, cilia are sensory organelles that are periodically reabsorbed before and rebuilt after cell division [51], while this resorption cycle has been linked to control of cell cycle checkpoints [4], [52]. Since the re-absorption of the primary cilium is prerequisite for cell division, reduced ciliary disassembly can cause a delay in cell division and therefore reduce the proliferative potential of a cell population under certain conditions. Our data suggest that inhibition of Plk1 might increase the number of ciliated cells in proliferative tissues or tumors, which could increase the negative effect on proliferation. Recently, Plk1 has been successfully explored as a target in cancer treatment based on its role as a mitotic inhibitor (reviewed in [22]). Whether Plk1 inhibitors also affect the number of ciliated cells, response to growth signals mediated through cilia, or the potential of ciliated cells to re-enter mitosis during tissue repair and in tumorigenesis has not been investigated, but is of interest based on our results.

Importantly, our study suggests a role for Plk1 in the pathogenesis of nephronophthisis and related ciliopathies. We demonstrate that Plk1 can phosphorylate NPHP1, the scaffold protein of the NPH protein complex, on a unique amino acid residue, T87. Overexpressed NPHP1 and Plk1 co-immunoprecipitate efficiently, although NPHP1 itself does not contain the conserved binding domain (S-pS/pT-P) usually required for the interaction with the polo-box domain of Plk1 [53]. This domain is present in several NPHP1 interacting proteins, e.g. in nephrocystin-4 (NPHP4) and the protein tyrosine kinase 2 (Pyk2). Suggestively, Pyk2 has previously been shown to interact directly with HEF1 [54], [55], the Aurora-A activator, again implying a possible larger functional complex relevant to nephronophthisis and ciliary disassembly. In contrast to the robust co-immunoprecipitation of proteins overexpressed in cultured cells and the clear co-immunoprecipitation of endogenous Plk1 with overexpressed Flag.NPHP1 it was not possible to detect co-immunoprecipitation of both endogenous proteins, probably because the intracellular pool of Plk1 and NPHP1 in the transition zone is extremely small, and beyond the limits of detection, or because the transient interaction of the kinase Plk1 with its substrate NPHP1 is only of very short duration. In the future, it will be interesting to assess the appearance of phosphorylated NPHP1 at the transition zone following manipulation of Plk1 activity and expression, but at present, there is no available phospho-specific antibody to T87 of NPHP1. Taken together, our findings identify Plk1 as a novel player within the NPH protein complex and NPHP1 as a novel Plk1 substrate. Interestingly, a novel recently identified NPH-related gene, Ataxin-10, has also been reported to be a substrate of Plk1, although in a mitotic function [56]. Beside the NPHPs, Plk1 might have additional targets within the NPH complex, within the transition zone proteins or the ciliary proteome. Further studies focused on the identification of ciliary substrates of Plk1 would help to understand the specific function of the ciliary Plk1 and its role in the pathogenesis of ciliopathies.

## Supporting Information

Figure S1 **Ciliated hTERT-RPE1 cells were stained with antibodies directed against either Plk1 (green) or phospho-Plk1 (green), acetylated α-tubulin (orange), and γ-tubulin (red), and treated with DAPI to visualize DNA (blue).** The panel shows cells in serum-starved conditions compared to 2 hours and 24 hours after serum induction. The panel showing cells at 24 hours includes staining of mitotic cells. The scale bar represents 5 µm.
(TIF)

Figure S2 **Ciliated hTERT-RPE1 cells were stained with antibody to NPHP1 (green), acetylated α-tubulin (orange), and γ-tubulin (red), and treated with DAPI to visualize DNA (blue).** The panel shows cells grown under serum-starved conditions (0h) compared to 2 hours and 24 hours after serum induction either treated with the Plk1 inhibitor BI 2536 compared to vehicle DMSO or transfected with siRNA to Plk1 compared to scrambled control siRNA (scr). The scale bar represents 10 µm.
(TIF)

Figure S3 **Western analysis of whole cell lysates prepared from hTERT-RPE1 grown under starved, ciliated conditions (0h) or at 2 hours and 24 hours following serum treatment, showing the expression levels of total Plk1 after treatment of the cells either with siRNA to Plk1 or scrambled siRNA (scr).**
(TIF)

Figure S4 **Results of mass spectrometry showing the phosphorylation of His.NPHP1-1-205 at residue T87 (upper graph) and the His.NPHP1-1-205 T87A mutant (lower graph) after in vitro kinase assay with active Plk1 was performed.** The precursor of the phosphopeptide was detected with a mass accuracy of 4.41 ppm. The phosphopeptide was identified by the Sequest algorithm, fulfilling the filter criteria (see materials and methods section). In this peptide there are four possible phosphorylation site. The phosphoRS algorithm calculates a confidence measure for the sites of phosphorylation. The first threonine returned the highest probability (56.6%). For the peptide containing an alanine residue instead of a threonine 30 high confident fragment spectra were detected. For this peptide no phosphorylated form was detected.
(TIF)

## References

[1] Wheatley DN, Wang AM, Strugnell GE (1996). Expression of primary cilia in mammalian cells.. Cell Biol Int.

[2] Hildebrandt F, Benzing T, Katsanis N (2011). Ciliopathies.. N Engl J Med.

[3] Santos N, Reiter JF (2008). Building it up and taking it down: the regulation of vertebrate ciliogenesis.. Dev Dyn.

[4] Plotnikova OV, Golemis EA, Pugacheva EN (2008). Cell cycle-dependent ciliogenesis and cancer.. Cancer Res.

[5] Bettencourt-Dias M, Hildebrandt F, Pellman D, Woods G, Godinho SA (2011). Centrosomes and cilia in human disease.. Trends Genet.

[6] Barr MM, Sternberg PW (1999). A polycystic kidney-disease gene homologue required for male mating behaviour in C. elegans.. Nature.

[7] Sang L, Miller JJ, Corbit KC, Giles RH, Brauer MJ (2011). Mapping the NPHP-JBTS-MKS protein network reveals ciliopathy disease genes and pathways.. Cell.

[8] Hildebrandt F, Attanasio M, Otto E (2009). Nephronophthisis: disease mechanisms of a ciliopathy.. J Am Soc Nephrol.

[9] Schermer B, Hopker K, Omran H, Ghenoiu C, Fliegauf M (2005). Phosphorylation by casein kinase 2 induces PACS-1 binding of nephrocystin and targeting to cilia.. EMBO J.

[10] Fliegauf M, Horvath J, von Schnakenburg C, Olbrich H, Muller D (2006). Nephrocystin specifically localizes to the transition zone of renal and respiratory cilia and photoreceptor connecting cilia.. J Am Soc Nephrol.

[11] Williams CL, Li C, Kida K, Inglis PN, Mohan S (2011). MKS and NPHP modules cooperate to establish basal body/transition zone membrane associations and ciliary gate function during ciliogenesis.. J Cell Biol.

[12] Jauregui AR, Nguyen KC, Hall DH, Barr MM (2008). The Caenorhabditis elegans nephrocystins act as global modifiers of cilium structure.. J Cell Biol.

[13] Habbig S, Bartram MP, Muller RU, Schwarz R, Andriopoulos N (2011). NPHP4, a cilia-associated protein, negatively regulates the Hippo pathway.. J Cell Biol.

[14] Dafinger C, Liebau MC, Elsayed SM, Hellenbroich Y, Boltshauser E (2011). Mutations in KIF7 link Joubert syndrome with Sonic Hedgehog signaling and microtubule dynamics.. J Clin Invest.

[15] Liebau MC, Hopker K, Muller RU, Schmedding I, Zank S (2011). Nephrocystin-4 regulates Pyk2-induced tyrosine phosphorylation of nephrocystin-1 to control targeting to monocilia.. J Biol Chem.

[16] Pugacheva EN, Jablonski SA, Hartman TR, Henske EP, Golemis EA (2007). HEF1-dependent Aurora A activation induces disassembly of the primary cilium.. Cell.

[17] Plotnikova OV, Pugacheva EN, Dunbrack RL, Golemis EA (2010). Rapid calcium-dependent activation of Aurora-A kinase.. Nat Commun.

[18] Plotnikova OV, Pugacheva EN, Golemis EA (2011). Aurora A kinase activity influences calcium signaling in kidney cells.. J Cell Biol.

[19] Archambault V, Glover DM (2009). Polo-like kinases: conservation and divergence in their functions and regulation.. Nat Rev Mol Cell Biol.

[20] Lens SM, Voest EE, Medema RH (2010). Shared and separate functions of polo-like kinases and aurora kinases in cancer.. Nat Rev Cancer.

[21] Petronczki M, Lenart P, Peters JM (2008). Polo on the Rise-from Mitotic Entry to Cytokinesis with Plk1.. Dev Cell.

[22] Strebhardt K (2010). Multifaceted polo-like kinases: drug targets and antitargets for cancer therapy.. Nat Rev Drug Discov.

[23] Johmura Y, Soung NK, Park JE, Yu LR, Zhou M (2011). Regulation of microtubule-based microtubule nucleation by mammalian polo-like kinase 1.. Proc Natl Acad Sci U S A.

[24] Schermer B, Ghenoiu C, Bartram M, Muller RU, Kotsis F (2006). The von Hippel-Lindau tumor suppressor protein controls ciliogenesis by orienting microtubule growth.. J Cell Biol.

[25] Otto EA, Schermer B, Obara T, O'Toole JF, Hiller KS (2003). Mutations in INVS encoding inversin cause nephronophthisis type 2, linking renal cystic disease to the function of primary cilia and left-right axis determination.. Nat Genet.

[26] Vazquez-Martin A, Oliveras-Ferraros C, Cufi S, Menendez JA (2011). Polo-like kinase 1 regulates activation of AMP-activated protein kinase (AMPK) at the mitotic apparatus.. Cell Cycle.

[27] Shevchenko A, Tomas H, Havlis J, Olsen JV, Mann M (2006). In-gel digestion for mass spectrometric characterization of proteins and proteomes.. Nat Protoc.

[28] Rappsilber J, Mann M, Ishihama Y (2007). Protocol for micro-purification, enrichment, pre-fractionation and storage of peptides for proteomics using StageTips.. Nat Protoc.

[29] Barr FA, Sillje HH, Nigg EA (2004). Polo-like kinases and the orchestration of cell division.. Nat Rev Mol Cell Biol.

[30] Benzing T, Schermer B (2011). Transition zone proteins and cilia dynamics.. Nat Genet.

[31] Nakajima H, Toyoshima-Morimoto F, Taniguchi E, Nishida E (2003). Identification of a consensus motif for Plk (Polo-like kinase) phosphorylation reveals Myt1 as a Plk1 substrate.. J Biol Chem.

[32] Soung NK, Park JE, Yu LR, Lee KH, Lee JM (2009). Plk1-dependent and -independent roles of an ODF2 splice variant, hCenexin1, at the centrosome of somatic cells.. Dev Cell.

[33] Macurek L, Lindqvist A, Lim D, Lampson MA, Klompmaker R (2008). Polo-like kinase-1 is activated by aurora A to promote checkpoint recovery.. Nature.

[34] De Luca M, Lavia P, Guarguaglini G (2006). A functional interplay between Aurora-A, Plk1 and TPX2 at spindle poles: Plk1 controls centrosomal localization of Aurora-A and TPX2 spindle association.. Cell Cycle.

[35] Hanisch A, Wehner A, Nigg EA, Sillje HH (2006). Different Plk1 functions show distinct dependencies on Polo-Box domain-mediated targeting.. Mol Biol Cell.

[36] van Leuken R, Clijsters L, van Zon W, Lim D, Yao X (2009). Polo-like kinase-1 controls Aurora A destruction by activating APC/C-Cdh1.. PLoS One.

[37] Kishi K, van Vugt MA, Okamoto K, Hayashi Y, Yaffe MB (2009). Functional dynamics of Polo-like kinase 1 at the centrosome.. Mol Cell Biol.

[38] Yuan K, Huang Y, Yao X (2011). Illumination of mitotic orchestra during cell division: a Polo view.. Cell Signal.

[39] Lane HA, Nigg EA (1996). Antibody microinjection reveals an essential role for human polo-like kinase 1 (Plk1) in the functional maturation of mitotic centrosomes.. J Cell Biol.

[40] Lens SM, Voest EE, Medema RH (2010). Shared and separate functions of polo-like kinases and aurora kinases in cancer.. Nat Rev Cancer.

[41] Rosenbaum JL, Witman GB (2002). Intraflagellar transport.. Nat Rev Mol Cell Biol.

[42] Satir P, Christensen ST (2007). Overview of structure and function of mammalian cilia.. Annu Rev Physiol.

[43] Jiang ST, Chiou YY, Wang E, Chien YL, Ho HH (2009). Essential role of nephrocystin in photoreceptor intraflagellar transport in mouse.. Hum Mol Genet.

[44] Craige B, Tsao CC, Diener DR, Hou Y, Lechtreck KF (2010). CEP290 tethers flagellar transition zone microtubules to the membrane and regulates flagellar protein content.. J Cell Biol.

[45] Cao M, Li G, Pan J (2009). Regulation of cilia assembly, disassembly, and length by protein phosphorylation.. Methods Cell Biol.

[46] Pan J, Naumann-Busch B, Wang L, Specht M, Scholz M (2011). Protein phosphorylation is a key event of flagellar disassembly revealed by analysis of flagellar phosphoproteins during flagellar shortening in Chlamydomonas.. J Proteome Res.

[47] Liu XS, Song B, Liu X (2010). The substrates of Plk1, beyond the functions in mitosis.. Protein Cell.

[48] Lowery DM, Clauser KR, Hjerrild M, Lim D, Alexander J (2007). Proteomic screen defines the Polo-box domain interactome and identifies Rock2 as a Plk1 substrate.. EMBO J.

[49] Santamaria A, Wang B, Elowe S, Malik R, Zhang F (2011). The Plk1-dependent phosphoproteome of the early mitotic spindle.. Mol Cell Proteomics 10: M110 004457.

[50] Grosstessner-Hain K, Hegemann B, Novatchkova M, Rameseder J, Joughin BA (2011). Quantitative phospho-proteomics to investigate the Polo-like kinase 1-dependent phospho-proteome.. Mol Cell Proteomics.

[51] Ishikawa H, Marshall WF (2011). Ciliogenesis: building the cell's antenna.. Nat Rev Mol Cell Biol.

[52] Kim S, Tsiokas L (2011). Cilia and cell cycle re-entry: more than a coincidence.. Cell Cycle.

[53] Elia AE, Cantley LC, Yaffe MB (2003). Proteomic screen finds pSer/pThr-binding domain localizing Plk1 to mitotic substrates.. Science.

[54] Manie SN, Beck AR, Astier A, Law SF, Canty T (1997). Involvement of p130(Cas) and p105(HEF1), a novel Cas-like docking protein, in a cytoskeleton-dependent signaling pathway initiated by ligation of integrin or antigen receptor on human B cells.. J Biol Chem.

[55] Astier A, Manie SN, Avraham H, Hirai H, Law SF (1997). The related adhesion focal tyrosine kinase differentially phosphorylates p130Cas and the Cas-like protein, p105HEF1.. J Biol Chem.

[56] Li J, Wang J, Hou W, Jing Z, Tian C (2011). Phosphorylation of Ataxin-10 by polo-like kinase 1 is required for cytokinesis.. Cell Cycle.

