# Supplementary figures and images for: The Centrosomal Kinase Plk1 Localizes to the Transition Zone of Primary Cilia and Induces Phosphorylation of Nephrocystin-1
Source: PLoS One. 2012 Jun 11;7(6):e38838. doi: 10.1371/journal.pone.0038838 (PMC3372538; doi:10.1371/journal.pone.0038838)

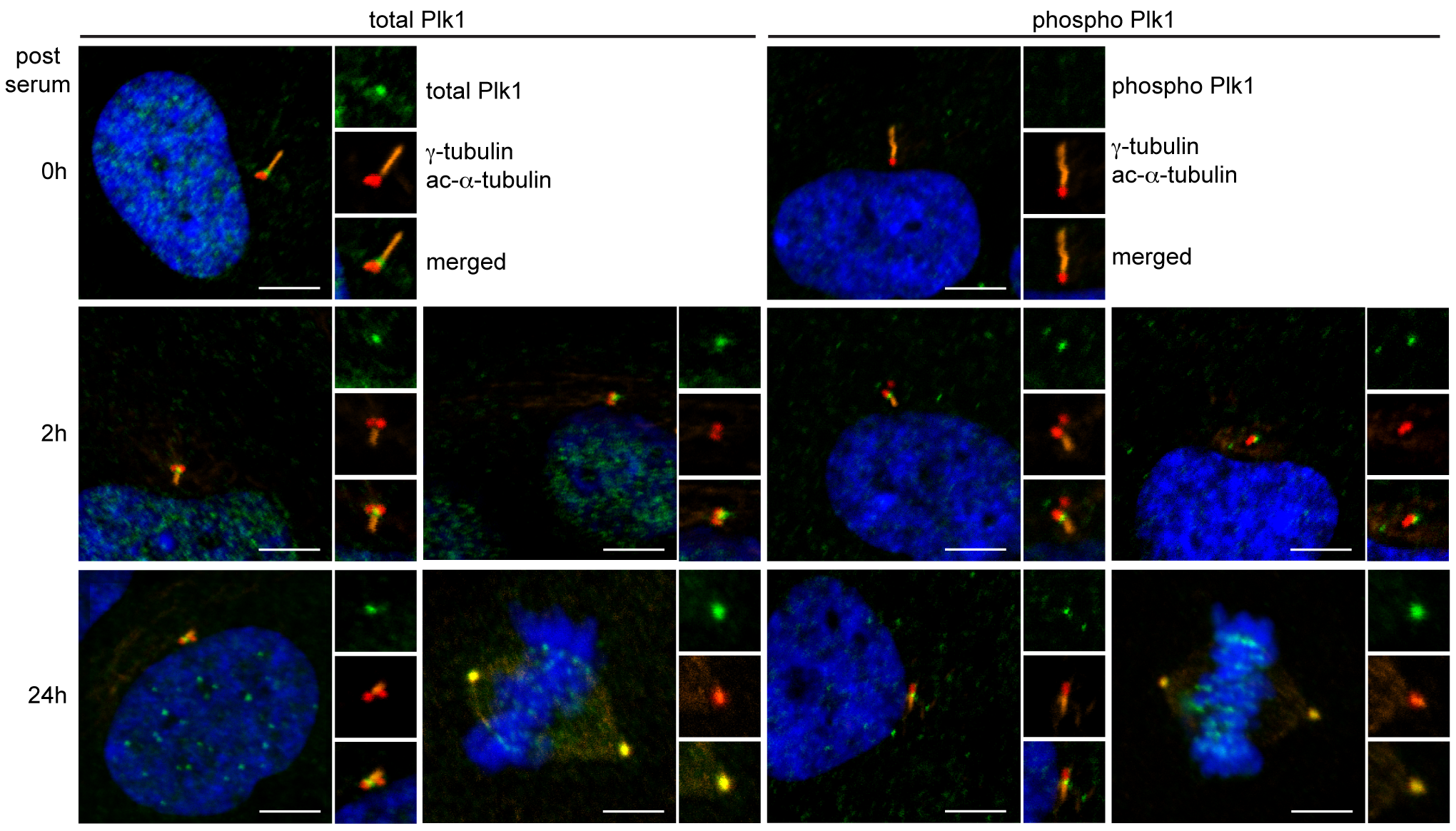

Supplement: Figure S1 — Ciliated hTERT-RPE1 cells were stained with antibodies directed against either Plk1 (green) or phospho-Plk1 (green), acetylated α-tubulin (orange), and γ-tubulin (red), and treated with DAPI to visualize DNA (blue). The panel shows cells in serum-starved conditions compared to 2 hours and 24 hours after serum induction. The panel showing cells at 24 hours includes staining of mitotic cells. The scale bar represents 5 µm. (TIF) [file pone.0038838.s001.tif]

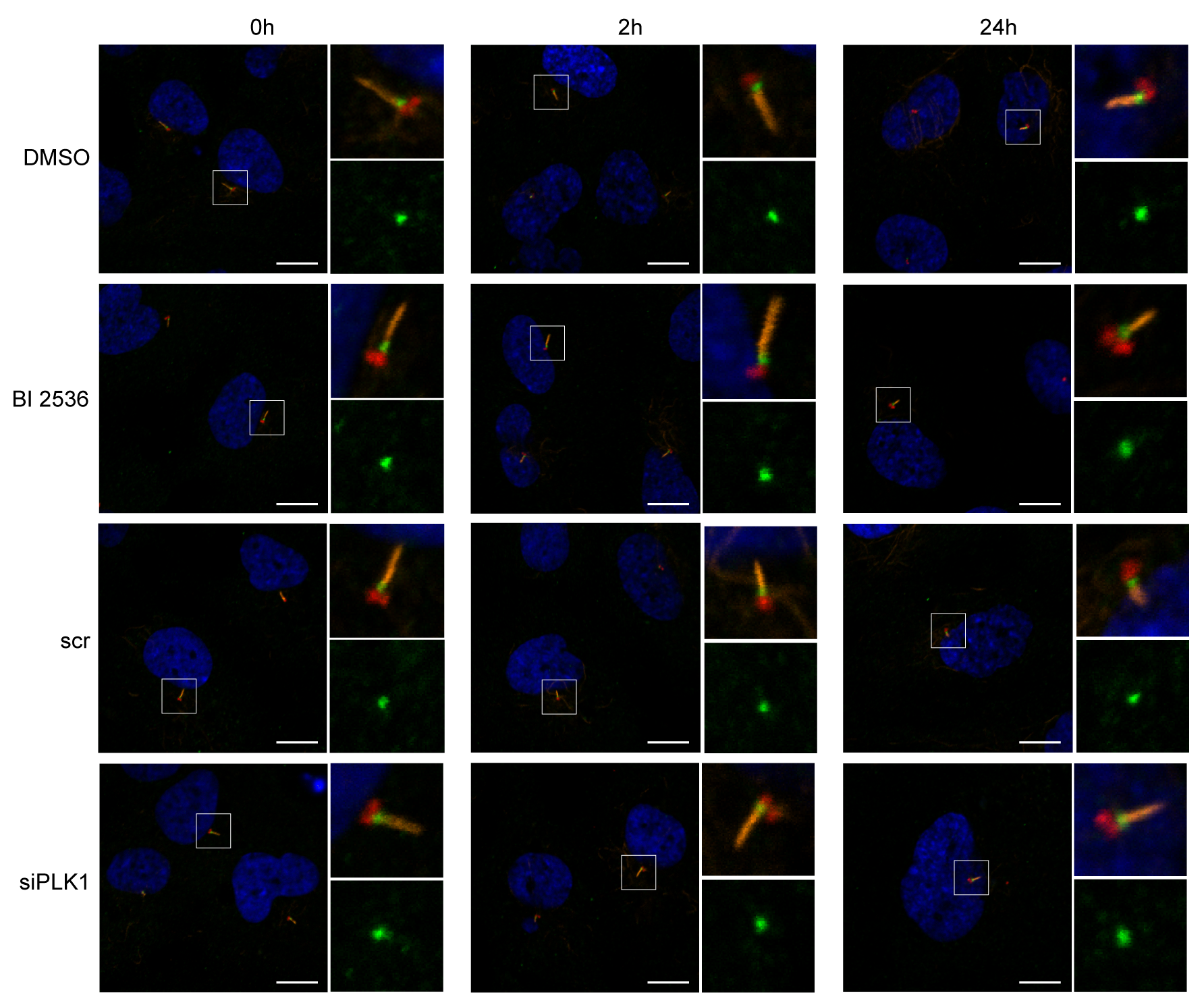

Supplement: Figure S2 — Ciliated hTERT-RPE1 cells were stained with antibody to NPHP1 (green), acetylated α-tubulin (orange), and γ-tubulin (red), and treated with DAPI to visualize DNA (blue). The panel shows cells grown under serum-starved conditions (0h) compared to 2 hours and 24 hours after serum induction either treated with the Plk1 inhibitor BI 2536 compared to vehicle DMSO or transfected with siRNA to Plk1 compared to scrambled control siRNA (scr). The scale bar represents 10 µm. (TIF) [file pone.0038838.s002.tif]

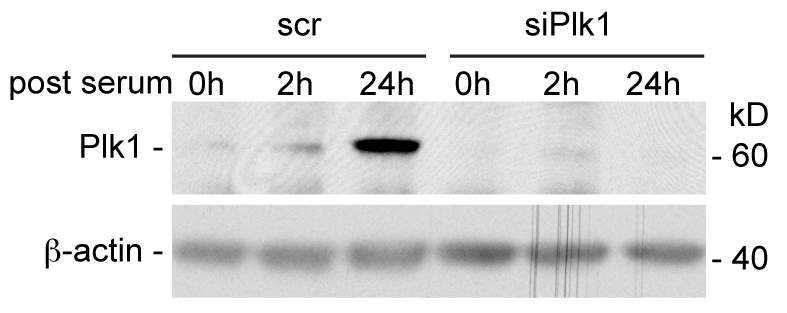

Supplement: Figure S3 — Western analysis of whole cell lysates prepared from hTERT-RPE1 grown under starved, ciliated conditions (0h) or at 2 hours and 24 hours following serum treatment, showing the expression levels of total Plk1 after treatment of the cells either with siRNA to Plk1 or scrambled siRNA (scr). (TIF) [file pone.0038838.s003.tif]

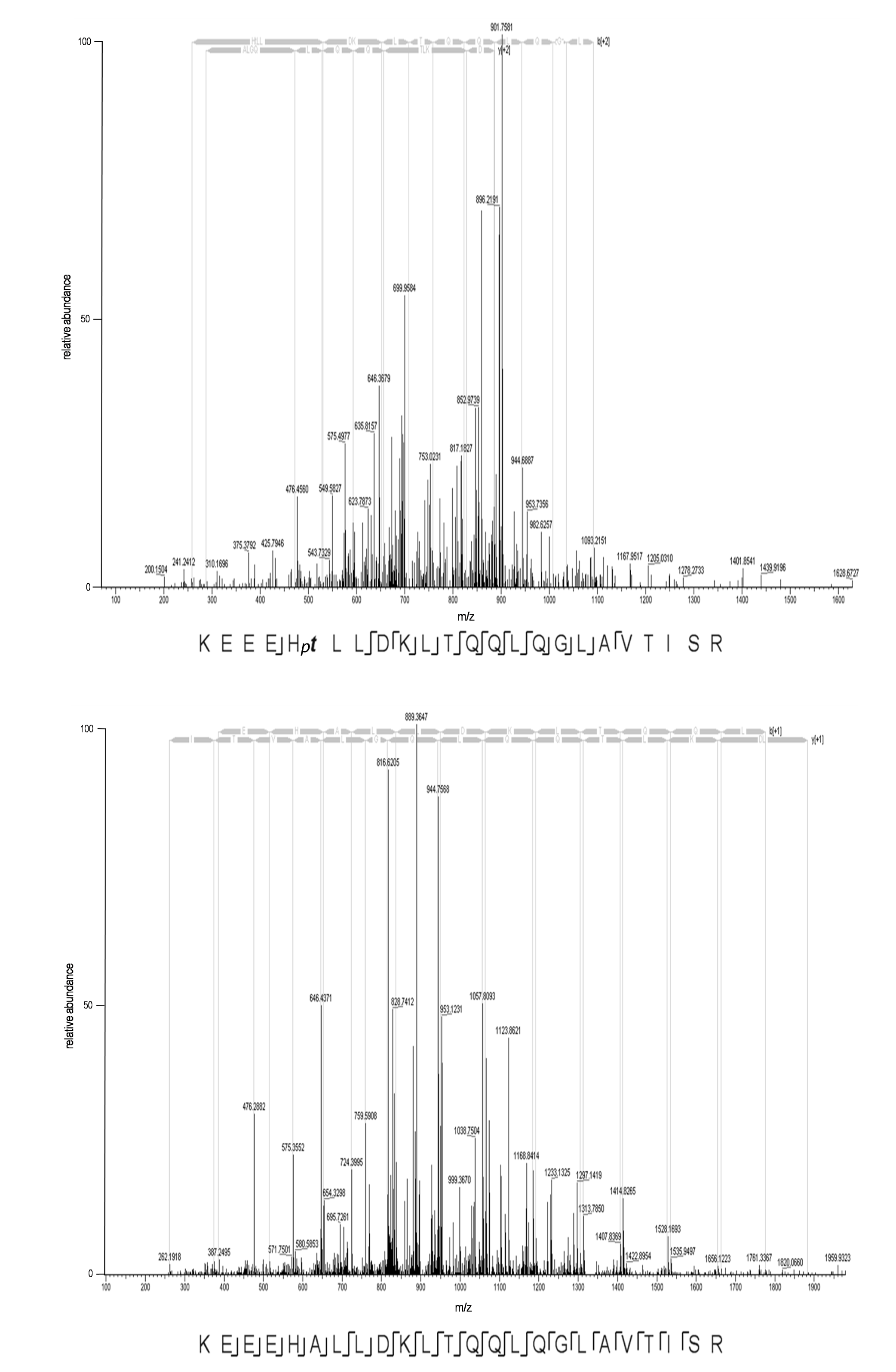

Supplement: Figure S4 — Results of mass spectrometry showing the phosphorylation of His.NPHP1-1-205 at residue T87 (upper graph) and the His.NPHP1-1-205 T87A mutant (lower graph) after in vitro kinase assay with active Plk1 was performed. The precursor of the phosphopeptide was detected with a mass accuracy of 4.41 ppm. The phosphopeptide was identified by the Sequest algorithm, fulfilling the filter criteria (see materials and methods section). In this peptide there are four possible phosphorylation site. The phosphoRS algorithm calculates a confidence measure for the sites of phosphorylation. The first threonine returned the highest probability (56.6%). For the peptide containing an alanine residue instead of a threonine 30 high confident fragment spectra were detected. For this peptide no phosphorylated form was detected. (TIF) [file pone.0038838.s004.tif]
